# Influence of sickle cell disease on susceptibility to HIV infection

**DOI:** 10.1371/journal.pone.0218880

**Published:** 2020-04-08

**Authors:** Shannon Kelly, Evan S. Jacobs, Mars Stone, Sheila M. Keating, Tzong-Hae Lee, Daniel Chafets, John Heitman, Melanie Dimapasoc, Eva Operskalski, Ward Hagar, Elliott Vichinsky, Michael P. Busch, Philip J. Norris, Brian Custer, for the Recipient Epidemiology and Donor Evaluation Study (REDS-III)

**Affiliations:** 1 Vitalant Research Institute, San Francisco, CA, United States of America; 2 UCSF Benioff Children’s Hospital Oakland, Oakland, CA, United States of America; 3 Keck School of Medicine, University of Southern California, Los Angeles, CA, United States of America; University of Texas Rio Grande Valley, UNITED STATES

## Abstract

People with sickle cell disease (SCD) are reported to have low rates of HIV infection, slower progression to AIDS and lower HIV-associated mortality compared to the general population. Mechanisms of potential resistance to HIV in SCD are incompletely understood. We retrospectively reviewed the Transfusion Safety Study to compare HIV status between people with SCD and other congenital anemias who were routinely exposed to blood products during the high-risk period before HIV screening implementation. Non-SCD congenital anemia diagnosis was associated with a higher risk of HIV acquisition compared to SCD (OR 13.1 95%CI 1.6–108.9). In addition, we prospectively enrolled 30 SCD cases and 30 non-SCD controls to investigate potential mechanisms of resistance to HIV in SCD. CCR5 and CCR7 expression was lower and CD4 expression was higher on CD4^+^ T cells from SCD cases compared to controls. Surface expression of CD4^+^ T cell CXCR4, CD38 and HLA-DR did not differ between the groups. SCD CD4^+^ T cells were not less susceptible to HIV infection than controls. Levels of multiple cytokines were elevated in the SCD plasma, but SCD plasma compared to control plasma did not inhibit HIV infection of target cells. In conclusion, our epidemiological data support people with SCD being resistant to HIV infection. Potential mechanisms include lower CD4^+^ T cell expression of CCR5 and CCR7, balanced by increased CD4 expression and cytokine levels, which did not result in *in vitro* resistance to HIV infection. Further study is needed to define the risk and pathophysiology of HIV in persons with SCD.

## Introduction

There is limited literature focused on HIV infection in sickle cell disease (SCD), but published reports suggest HIV is relatively rare and disease penetrance is low in this population. Most studies comparing HIV prevalence in SCD to a non-SCD population have shown lower rates of HIV in SCD (literature summarized in **supporting information Table A in S1 File**). [1–5]. Comparatively low HIV-associated mortality and progression to AIDS in SCD patients have also been reported [6–8]. Speculation on the underlying mechanisms for lower HIV prevalence and/or progression in SCD has included behavioral factors reducing exposure and an inhibition of HIV replication due to changes in iron metabolism or the pro-inflammatory component of SCD pathophysiology [9].

In this study we sought to bring different lines of evidence together to confirm the lower rate of HIV infection in SCD and to explore specific mechanisms of resistance to HIV in this population. First, to determine if we could replicate epidemiologic data suggesting a lower prevalence of HIV in SCD, we retrospectively reviewed data from the Transfusion Safety Study (TSS). The TSS was an NHLBI multicenter study conducted from the1980’s through 1993 designed to investigate factors influencing the occurrence of retrovirus infections among persons receiving blood components prior to HIV screening in 1985 [10]. Second, we prospectively enrolled SCD patients and non-SCD controls into a study to investigate multiple potential mechanisms of HIV infection resistance in SCD.

## Materials and methods

### Retrospective analysis of the transfusion safety study

The TSS has been described in detail [10]. Briefly, transfused subjects and non-transfused controls were enrolled in four areas of, at the time, high AIDS prevalence (New York, Miami, San Francisco and Los Angeles) and two areas of low prevalence (Detroit and Seattle) beginning in 1985 with follow-up observations through 1993. Subjects were screened by enzyme immunoassay, with positivity confirmed by western blot, to determine HIV status at study entry and were retested every 6 months. Included among the study groups were persons with congenital anemias (SCD, thalassemia and Diamond Blackfan Anemia [DBA]) or clotting disorders who were routinely exposed to blood products. Patients with clotting disorders were excluded from this analysis as they received pooled factor concentrate from many donors, and therefore were exposed to a much higher risk product than congenital anemia patients who were exposed to single donor products. The HIV prevalence at study entry was compared between SCD and other congenital anemia patients (thalassemia/DBA). There were no look-back investigation policies in place at the time and HIV could not be proven to be transfusion-transmitted. However, many children with no other HIV risk factors were included in the study population and overall HIV prevalence is assumed to be a surrogate for transfusion transmitted HIV.

### Prospectively studied subjects

Consecutive patients with SCD who were >18 years of age were recruited at routine visits at the adult SCD clinic at UCSF Benioff Children’s Hospital Oakland (BCHO) in 2014. Controls frequency matched on gender, race and age within 5 years of enrolled SCD patients were recruited from the blood donor population at Blood Centers of the Pacific, San Francisco, during the same time period. Subjects were eligible if there was no treatment with nonsteroidal anti-inflammatory drugs in the previous 1 day, steroid treatment in the previous 14 days, acute visit or hospitalization within the previous 14 days, blood transfusion in the previous 8 weeks or co-existing auto-immune or inflammatory disorders. Patients were required to be HIV negative by any HIV test used for screening as part of clinical care within the previous 5 years. All patients and controls were confirmed to be HIV negative using the OraQuick HIV test upon enrollment. Initial eligibility criteria for patients were restricted to homozygous SS type SCD and no hydroxyurea (HU) treatment. However, with high prevalence of HU treatment, blood transfusions and NSAIDs in the SCD population, identification of eligible patients was difficult. Therefore eligibility criteria were expanded to include all SCD genotypes and HU treatment. The study was approved by the BCHO institutional review board and all participants signed informed consent.

#### Sample collection

Thirty SCD patients and 30 non-SCD control subjects had approximately 30 mL whole blood collected in EDTA tubes. Peripheral blood mononuclear cells (PBMCs) were isolated by ficoll, frozen, and maintained in liquid nitrogen, and separated plasma was stored at -80°C.

#### Immunophenotyping

Patient/control whole blood was analyzed for HIV co-receptors and activation state of PBMCs. Samples were labeled with an antibody cocktail containing: CD3-PE (Clone HIT3a), CD4-Alexa700 (RPA-T4), CD8-APC-Cy7 (SK1), CD38-BV421 (HIT2), HLA-DR-FITC (L243), CCR5-Alexa647 (HEK/1/85a), CCR7-PECy7 (G043H7), and CXCR4-PerCP-Cy5.5 (12G5) (all from Biolegend) at room temperature for 30 minutes. Following labeling, 2 mL FACS lysing solution was added to the samples and they were incubated for an additional 10 minutes at room temperature and then pelleted, washed in PBS, fixed in 1% paraformaldehyde and acquired on a BD LSRII (BD Biosciences). Data were analyzed with FlowJo software (TreeStar).

#### Viruses, cells, and reagents

Clonal virus stocks were generated by transfection of 4 x 10^6^ 293T cells with 10 μg of plasmid DNA from HIV molecular clones NL4-3 and 81.A. Transfections were carried out using Fugene 6 (Roche) at a ratio of 1.5 μL Fugene per 1 μg DNA according to the manufacturer’s directions. Culture supernatants were harvested 48 hours post infection, centrifuged to remove cell debris, aliquoted, and stored at -80°C until use. The TCID_50_ of each virus stock was determined in MT-2-CCR5^hi^ cells (provided by Dr. Donald Mosier). MT-2-CCR5^hi^ cells were maintained at log phase in RPMI 1640 media (UCSF Cell Culture Facility (CCF)) supplemented with 20% heat-inactivated fetal calf serum (Hyclone), 12 mM HEPES (UCSF-CCF) and penicillin/streptomycin (UCSF-CCF) (R20).

#### Multiplex cytokine analysis

Serum samples were assayed using the following: High-sensitivity Milliplex Map kit (Millipore) for interleukin(IL)-1β, IL-2, IL-4, IL-5, IL-6, IL-7, IL-8, IL-10, IL-12 p70, IL-13, interferon (IFN)-γ, granulocyte macrophage stimulating factor (GM-CSF) and tumor necrosis factor (TNF)-α; Standard sensitivity Milliplex Map kit (Millipore) for endothelial growth factor (EGF), fibroblast growth factor (FGF)-2, fractalkine, IL-1α, IL-1 receptor antagonist (Ra), IL-9, IL-12(p40), IL-15, IL-17, interferon induced protein (IP)-10, monocyte chemotactic protein (MCP)-1, MCP-3, monocyte derived chemokine (MDC), macrophage inflammatory protein (MIP)-1α, MIP-1β, sIL-2 receptor a, TNF-β, and VEGF; Standard Sensitivity Panel II kit (Millipore) for CCL8/MCP-2, CCL13/MCP-4, CXCL5/ human neutrophil-activating peptide (ENA)-78, CXCL12/stromal derived factor (SDF)-1α+β, CXCL13/ B-cell attracting chemokine (BCA)-1, CCL1/I-309, IL-16, CCL15/MIP-1δ, CCL17/thymus and activation regulated chemokine (TARC), CCL21/6Ckine, CCL24/Eotaxin-2, CCL26/Eotaxin-3, CCL27/cutaneous T-cell attracting chemokine (CTACK), IL-23, leukemia inhibitory factor (LIF), thrombopoiesis stimulating factor (TPO), TNF-related apoptosis inducing ligand (TRAIL), stromal cell-derived *cytokine* (SCF), thymic stromal lymphopoietin (TSLP), IL-20, IL-21, IL-28A, and IL-33; Standard Sensitivity Panel III kit (Millipore) for macrophage colony stimulating factor (M-CSF), CXCL9/monokine induced by gamma (MIG), neutrophil attractant protein (NAP), CXCL6/ granulocyte chemotactic protein (GCP)-2, CXCL11/ Interferon-inducible T cell alpha chemoattractant (I-TAC), hemofiltrate CC-Chemokine (HCC)-1, CXCL19/MIP-3β, CCL20, MIP-3α, CL1/Lymphotactin, IL-11, and IL-29/IFN-λ. Standards and samples were tested in duplicate. Beads were acquired on a Luminex LX200 (Austin, TX) and analyzed using Bio-Plex manager 6.1 software and Bio-Plex Data Pro (Bio-Rad).

#### Infection and virus culture assay

As described previously, PBMCs were depleted of CD8^+^ T cells via CD8 positive selection kits (Stem Cell Technologies) and infected with CXCR4 (X4)-tropic virus (NL4-3) or CCR5 (R5)-tropic virus (81-A) at a multiplicity of infection (MOI) of 10^−2^,10^−3^, and 10^−4^ for 2 hrs[11]. Following infection, cells were washed and seeded into 96-well culture dishes at 1x10^6^ cell/mL in R20 with 50 IU/mL rhIL-2. On Day 3 a 50% media exchange was performed, and the cells were cultured for 3 more days. Following culture, cell viability was determined with acridine orange and propidium iodide labeling using the Auto X4 cell counter (Nexcelom Bioscience). Supernatants were harvested and maintained at -80°C until analysis for HIV p24 by ELISA according to manufacturer’s instructions (Applied Bioscience Laboratories).

MT-2 cells in log phase growth were infected in the presence or absence of patient or control plasma. MT-2 cells were aliquoted at 5x10^4^ cells/well into 96-well culture dishes with 20% patient or control plasma and infected at an MOI of 10^−2^ with HIV 81-A or NL4-3. Infected cultures were maintained for four days. On day 4 wells were mixed and half the volume was removed and replaced with fresh media and 20% patient or control plasma and culture was continued for three more days. Following seven day culture, supernatants were stored at -80°C until analysis for HIV p24 by ELISA.

#### Measurement of HIV proviral DNA

HIV proviral DNA was quantified as previously described[12]. Following virus culture assay, infected cells were washed twice with Tris lysis buffer solution A (UCSF-CCF), and then resuspended in lysis buffer (100 μL Tris buffer A, 100 μL Tris buffer B, 1.25 μL proteinase K (Qiagen) for each sample), covered with a plastic plate cover and heated for 1.5 hrs at 60°C with gentle vortexing every 20 minutes. Samples were subsequently heated to 95°C for 30 minutes to inactivate proteinase K. For a 115 base pair HIV DNA amplification, the following primers were used: SK38 (5’- ATA ATC CAC CTA TCC CAG TAG GAG AAA T -3’) and SK39 (5’- TTT GGT CCT TGT CTT ATG TCC AGA ATG C -3’). For a 242 base pair region of HLA-DQ amplification the following primers were used: GH26 (GTGCTGCAGGTGTAAACTTGTACCAG) and GH27 (CACGGATCCGGTAGCAGCGGTAGAGTTG). A total of 5 μL of the DNA lysate was added to 10 μL of PCR mixture consisting of 1 mM of dNTPs (Roche Diagnostics GMBH), 1 uM of each primer (Integrated DNA Technologies), SYBR Green (3.75 units/reaction; BioWhittaker Molecular Applications), and FastStart Taq (0.7 units/reaction; Roche Diagnostics). Real-time PCR was performed using the Roche 480 (Roche Diagnostics) with cycle conditions 1 minute at 95°C followed by 45 cycles of 30 seconds at 95°C, 30 seconds at 64°C, and 45 seconds at 72°C. Each sample was tested in two or three replicate reactions. Relative proviral load was defined as the ratio of HIV compared to HLA-DQα transcripts, expressed as a percentage.

#### Measurement of integrated HIV

Integrated HIV was measured as described by Vandergeeten et al[13]. Briefly, infected cells were digested with proteinase K, and the lysates were used in a preamplification reaction to coamplify integrated HIV DNA (using primers targeting the HIV LTR and Alu elements within the human genome) together with human CD3 gene sequences (to allow quantification of cellular input). The products of the preamplification reaction were then used for real-time PCR amplification with primers and probes targeting either the preamplified HIV DNA or the human CD3 gene, together with 0.8 mM dNTPs (Bioline) and AmpliTaq Gold (0.95 units/reaction; Life Technologies). Reactions were performed on a LightCycler 480 instrument (Roche) with the following cycle conditions: denaturation for 10 min at 95°C, followed by 45 cycles of amplification (15 sec at 95°C, 1 min at 60°C). Each sample was tested in two or three replicate reactions. Results were expressed as integrated HIV DNA copies per 10^6^ cells.

### Statistical analysis

Baseline characteristics of congenital anemia subjects who were HIV seropositive and seronegative at TSS entry were compared in univariate analysis using chi-square or Fisher’s exact tests. Multivariable logistic regression models were then used to determine if the type of congenital anemia was associated with odds of HIV acquisition after controlling for factors which demonstrated a p value <0.1 in univariate analysis.

Graphical representations of immunophenotyping, infectivity data and cytokine data were prepared using Prism (GraphPad). Differences were measured using a two-tailed Student’s T test for normally distributed data and a Wilcoxon-Mann-Whitney test for non-parametric data. As frequency matching was conducted during enrollment, paired statistical tests were not used. To account for multiple comparisons for cytokines, the multitest procedure (SAS 9.3) was used to provide linear step-up false discovery rate (FDR) p-value adjustments for cytokine comparisons by the method of Benjamani and Hochberg[14]. For all analyses, a two-sided p value of <0.05 was considered significant.

## Results

### Retrospective analysis of the Transfusion Safety Study (TSS)

SCD patients in the TSS were compared to contemporary patients receiving transfusions for other congenital anemias. Of the 274 analyzed congenital anemia patients enrolled in TSS with documented transfusion from 1981 to study enrollment, 21 (7.7%) were HIV seropositive at study entry. There was a lower prevalence of HIV in SCD subjects (1 of 143, 0.7%) compared to subjects with thalassemia or DBA (20 of 131, 15.3%, p<0.0001). The number of transfused units and location were also associated with HIV positivity. The association of gender was borderline significant, and there was no association with age (**Table 1**). Multivariable logistic regression showed thalassemia/DBA was associated with a higher risk of HIV acquisition compared to SCD after controlling for gender and number of units transfused (OR13.1 [1.6–108.9]), **Table 2**). Location could not be included in the multivariable model as no patients in the low HIV prevalence locations were HIV+. There were more thalassemia/DBA patients in high prevalence locations (127 of 131, 97%) compared to SCD patients (88 of 143, 61.5%). However, in a multivariable model restricted to include only patients in high prevalence locations, similar results were seen with higher odds of HIV with thalassemia/DBA compared to SCD (OR 6.7 [0.7–60.5, p = 0.09). None of the enrolled congenital anemia patients seroconverted to HIV positivity during the follow-up period after implementation of HIV screening of blood donors in 1985.

**Table 1:** Baseline characteristics of participants in the transfusion safety study at study entry, by HIV status.

	HIV Positive n = 21	HIV Negative n = 253	Total n = 274	P value*
Disease				<0.0001
SCD	1 (0.7%)	142 (99.3%)	143	
Thalassemia/DBA	20 (15.3%)	111 (84.7%)	131	
Gender				
Female	16 (10.5%)	136 (89.5%)	152	0.05
Male	5 (4.1%)	117 (95.9%)	122	
Age				
<13 years	7 (7.5%)	86 (92.5%)	93	0.43
13–24 years	11 (9.7%)	102 (90.3%)	113	
25+ years	3 (4.4%)	65 (96.6%)	68	
Transfusion Exposure**				0.0006
<18 units	1 (1.5%)	67 (98.5%)	68	
18–78 units	1 (1.5%)	68 (98.5%)	69	
79–142 units	12 (17.4%)	57 (82.6%)	69	
143+ units	7 (10.3%))	61 (89.7%)	68	
Site				0.01
High prevalence	21 (9.7%)	194 (90.2%)	215	
Low prevalence	0 (0%)	59 (100%)	59	

* Chi-square or Fisher’s exact test

**Number of units received from 1981 through study entry. Categories are quartiles.

**Table 2:** Odds of HIV Acquisition in TSS.

	OR [95%CI]*	p-value
Type of Anemia		0.02
SCD	1.0	
Thalassemia/DBA	13.1 [1.6–108.9]	
Gender		0.17
Male	1.0	
Female	2.1 [0.7–6.3]	
Blood components 1981-entry (units)		0.23
<18	1.0	
18–78	0.7 (0.04–11.4)	0.77
79–142	4.1(0.5–36.9)	0.20
143+	2.5 (0.3–23.8)	0.42

*OR = Odds ratio with 95% confidence interval is the odds of HIV acquisition compared to base category (shown with OR of 1).

### Prospectively enrolled subjects

There were 30 SCD patients and 30 non-SCD controls enrolled in the study. All participants were African-American. There were more male and SS genotype patients compared to female and SC/Sβ-thalassemia patients enrolled, and 37% were treated with HU (**Table 3**).

**Table 3:** Demographics of 30 SCD Subjects Prospectively Enrolled to Investigate Mechanisms of Resistance to HIV.

	SCD Cases (n = 30)	Non-SCD Controls (n = 30)
Demographic*	Mean (range) or N (%)	
Age (years)	39.2 (23–58 years)	39.4 (25–62)
Gender		
Male	20 (66.7%)	20 (66.7%)
Female	10 (33.3%)	10 (33.3%)
Race		
African American	30 (100%)	30 (100%)
Sickle Cell Type		
SS	17 (57%)	N/A
SC	11 (36%)	
Sβ+	2 (7%)	
Hydroxyurea		
Yes	11 (37%)	
No	19 (63%)	

*no significant differences between demographics of cases and controls

### Cellular markers relevant for HIV infection: HIV co-receptor expression, activation phenotype of CD4^+^ T cells and frequency and intensity of CD4 expression

**Fig 1:**
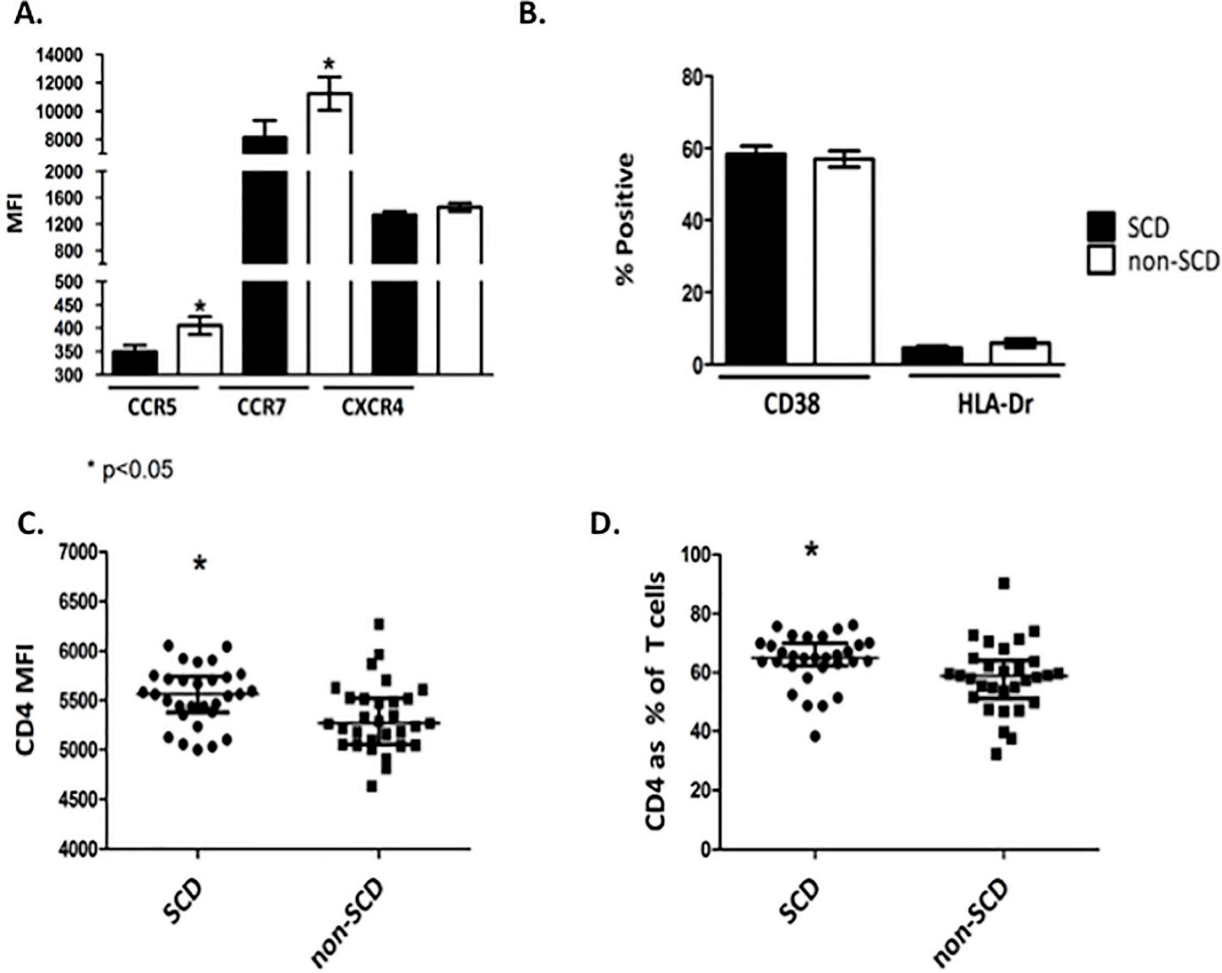
Comparison of HIV co-receptors, CD4+ T cell activation markers, CD4+ MFI and Percent of CD4+ Cells Among Total T Cells between SCD patients and non-SCD controls. Twenty four-hour old whole blood samples from SCD patient and non-SCD controls were labeled for CD4 T cells and incubated with antibodies to detect markers associated with HIV or cellular activation. Following incubation, surface expression of the HIV co-receptors CCR5 and CXCR4 and T cell trafficking molecule CCR7 were measured by flow cytometry (**Panel A**). MFI, mean fluorescence intensity between SCD patients and non-SCD controls were compared. Percent of CD4^+^ T cells expressing the activation markers CD38 or HLA-DR were measured and compared between SCD patients and non-SCD controls (**Panel B**). The intensity of expression of CD4+ amongst T cells (**Panel C**) and the percent of CD4+ T cells among total T cells (**Panel D**) was significantly higher in SCD patients compared to non-SCD controls. Mean and standard errors of the means for 30 SCD patients and 30 non-SCD controls are shown, *p<0.05.

We hypothesized that the lower risk of HIV acquisition seen in SCD patients might be due to lower expression of surface HIV co-receptors or decreased activation of CD4^+^ T cells. We found a significant decrease in the expression of the HIV co-receptor CCR5 in the SCD patients vs. controls (mean MFI 349.0 vs. 405.4 respectively, p<0.05; **Fig 1A**) but no difference in the expression of the other main HIV co-receptor, CXCR4, between the two groups (median MFI of 1340.9 vs. 1457.2; **Fig 1A**). There was decreased expression of the T cell trafficking molecule CCR7 in SCD patients compared to controls (mean MFI 8135.8 vs. 11229.7 respectively, <0.05; **Fig 1A**). There was no difference between patients and controls in the proportion of CD4^+^ T cells expressing the activation markers CD38 or HLA-DR (**Fig 1B**).

SCD patients were included whether or not they received hydroxyurea (HU). Given that HU has immunomodulatory properties[15], the expression of HIV co-receptors and markers of activation were compared in patients with or without HU treatment (**S1 Fig**). There were no significant differences observed between HU treated and not for CCR5, CCR7, CXCR4, and CD38 expression. Consistent with an anti-inflammatory role, there was a significantly lower proportion of HLA-DR positive cells in patients receiving HU (2.7 vs. 5.6% positive, p<0.05).

The lower levels of CCR5 expression might result in a lower susceptibility to HIV infection. However, there is an interdependence between CCR5 and CD4 regarding HIV infection, and higher levels of one receptor can compensate for lower levels of the other [16]. Considering this, the expression of CD4 amongst the PBMC populations was measured in the SCD patients and controls. There was a significant increase in CD4 expression in SCD patient PBMC compared to controls (mean MFI of 5545 vs. 5324; **Fig 1C**). We also examined the percentage of CD4^+^ T cells as a fraction of total T cells. Again, there was a significant increase in CD4^+^ T cells in SCD patients compared to controls (median of 64.2 vs. 58.1%; **Fig 1D**). These data demonstrate a higher percentage of CD4^+^ T cells in the SCD patient samples, providing more targets for infection and a higher level of CD4 expression on individual T cells.

### Susceptibility of SCD CD4^+^ T cells to HIV infection

**Fig 2:**
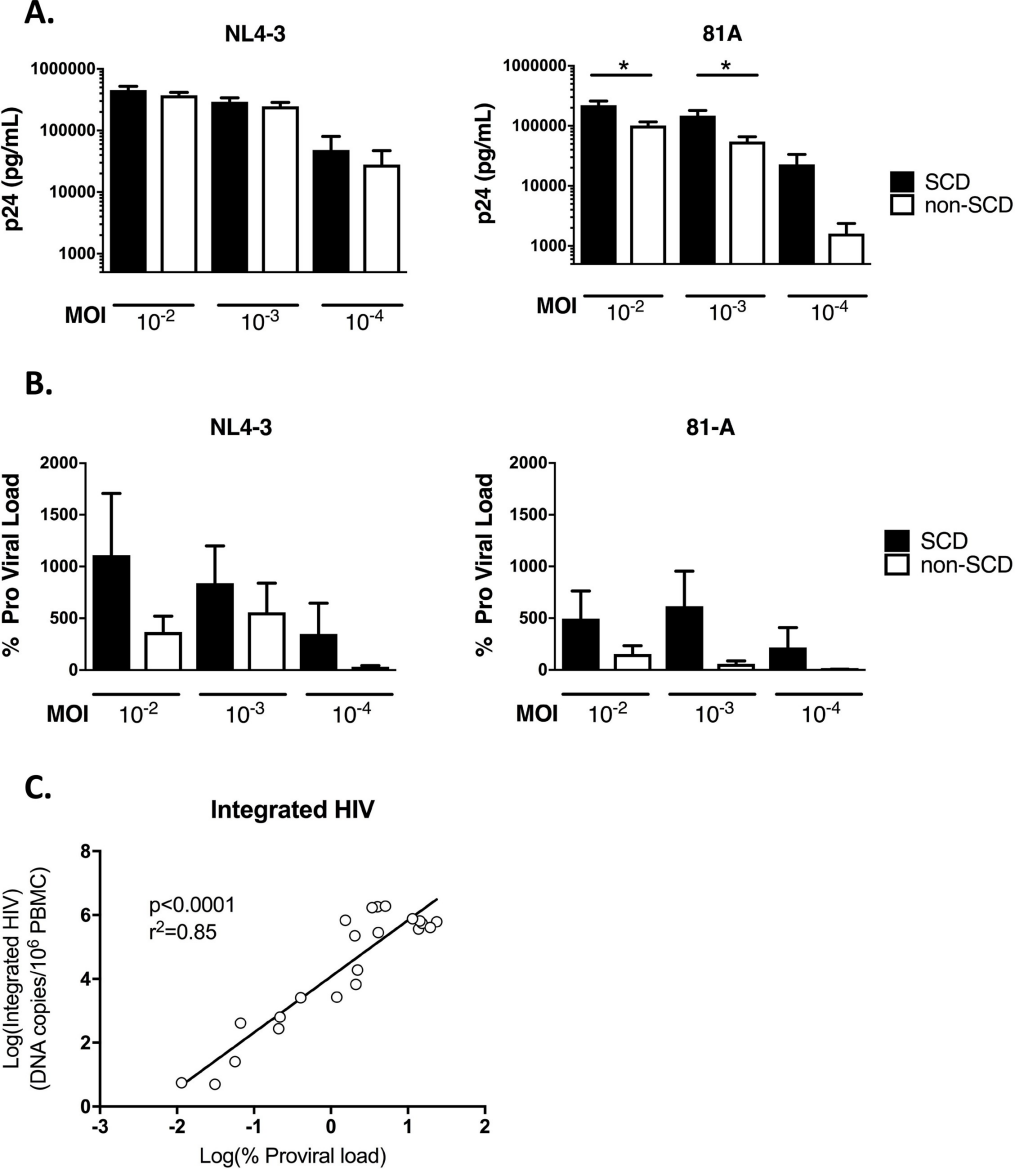
HIV Infectivity of PBMCs from SCD Patients and non-SCD Controls. CD8-depleted PBMC from SCD patients and non-SCD controls were infected with HIV NL4-3 (CXCR4-tropic) and 81-A (CCR5-tropic) at increasing MOI for six days. Following incubation, supernatants and cells were harvested for detection of HIV p24 (**Panel A**) and pro-viral load (**Panel B**) respectively. Mean and standard errors of the means for 30 SCD patients and 30 non-SCD controls are shown. Samples from four SCD patients and two non-SCD controls spanning a range of proviral load were assayed for integrated HIV DNA (**Panel C**). Five samples showed undetectable HIV DNA on both assays and are not graphed. The linear regression line of the log_10_ values is shown.

We next sought to determine if CD4^+^ T cells from SCD patients were less susceptible to HIV infection. Given the lower expression of CCR5, we hypothesized that SCD patient T cells would be less susceptible to infection with a CCR5-tropic strain of HIV. PBMCs from SCD patients and controls were infected with HIV using the X4 virus NL4-3 and the R5 virus 81-A at three virus concentrations. After X4 virus infection there were minimal differences in the production of HIV p24 six days post-infection (**Fig 2A**). Unexpectedly, when PBMC were infected with R5 virus, there was a small but significant increase in p24 production at the higher MOIs of 10^−2^ and 10^−3^ and a nonsignificant increase in p24 production at MOI 10^−4^ in SCD patient samples compared to controls (**Fig 2A**).

As with the phenotyping studies, the data were examined to determine if HU treatment influenced results. For both X4 and R5 viruses, subjects on HU treatment showed marginally lower p24 production after infection of their PBMCs, and this change was only significant for the MOI 10^−3^ infection with X4 virus (**S2 Fig**).

Although p24 is a measure for production of virus particles, many non-infectious particles result from HIV replication which cannot be differentiated by p24 measurement. Therefore, we also measured incorporation of HIV provirus in the cells of our *in vitro* infections, which is indicative of a productive viral infection. Similar to the p24 data, there was no significant difference in incorporated provirus of the SCD patients vs. controls (**Fig 2B**). In fact, there was a trend to increased incorporated provirus in SCD. The assay used to detect provirus could also detect 2-LTR circles, generated prior to viral integration. Therefore samples selected to span a range of proviral loads were selected to test for the presence of integrated HIV in infected target cells using a recently published method[13]. Five samples were negative for detectable provirus and were also negative for integrated HIV. The remaining samples from both R5 and X4 virus infection showed detectable integrated HIV, with close correlation to the HIV proviral load (**Fig 2C**). These data indicate that despite lower expression of CCR5, SCD patient CD4^+^ T cells are not less susceptible to infection with HIV and may be marginally more susceptible to infection in these *in vitro* experiments.

### Cytokine profiles in SCD

**Fig 3:**
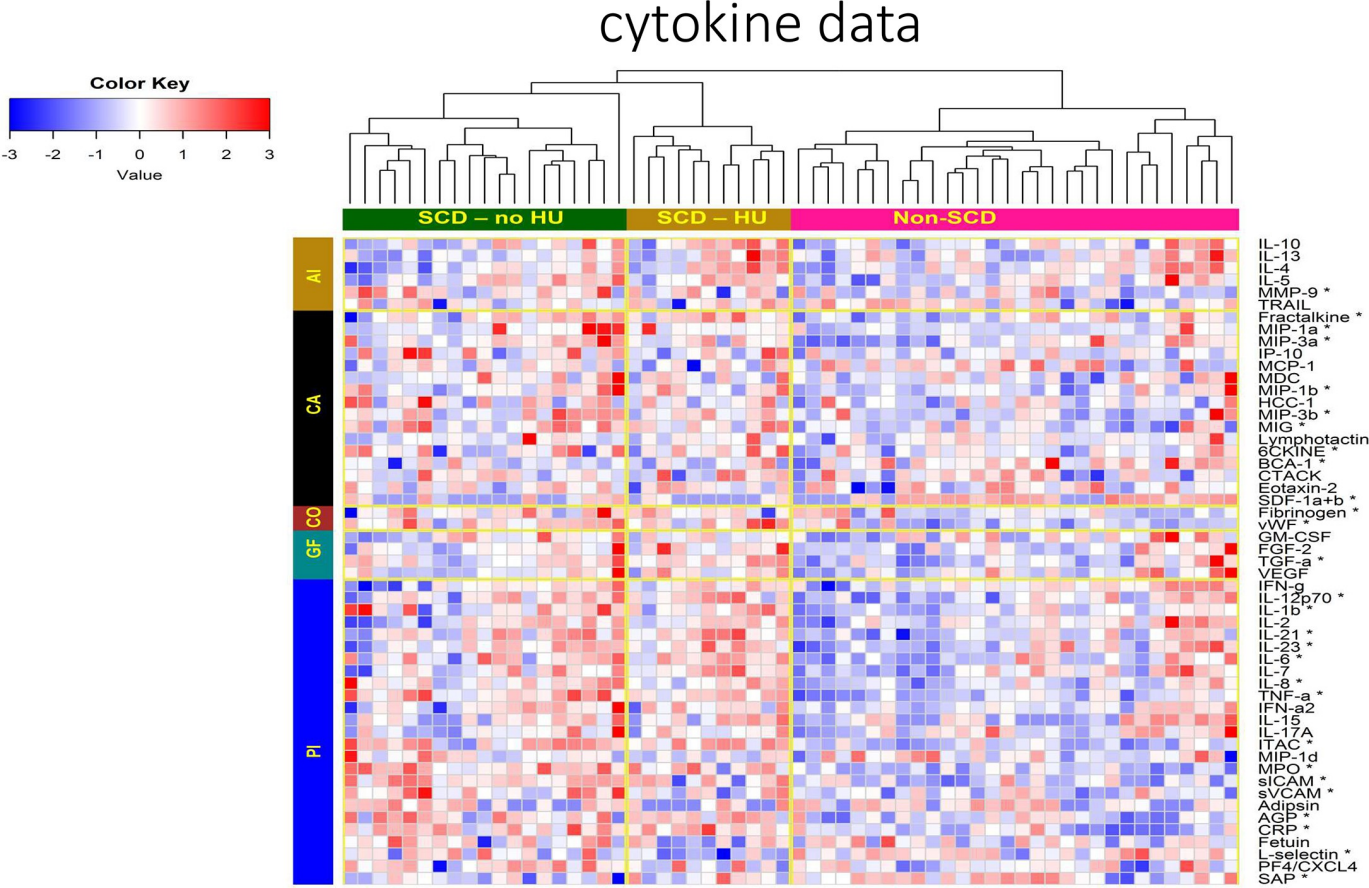
Cytokines and Chemokines Compared between SCD Patients (treated or not treated with HU) and non-SCD Controls. Biomarkers tested are grouped into anti-inflammatory (AI), chemoattractant (CA), coagulation (CO), growth factor (GF) and pro-inflammatory (PI) functional categories. Relative increases in concentration are shown in red and decreases in blue. * significantly different between SCD patients and non-SCD controls (p<0.05 after FDR correction).

In addition to the investigated cellular markers, plasma biomarkers were compared between SCD and non-SCD controls as innate antiviral immune responses including cytokines or receptor blocking chemokines present during exposure may play a role in preventing HIV infection. All cytokines tested are shown in **Fig 3**, (median and ranges of all measurements included in **Table B of S1 File**). A subset of inflammatory cytokines (TNF-α, IL-1β, IL-6, IL-8, IL-12p70, IL-21, IL-23, MPO, sICAM, sVCAM, CRP) and chemokines (MIP-1α, MIP-1β, ITAC, MIP-3α, MIP-3β, and 6CKINE) was significantly elevated in SCD compared to controls (Table B in **S1 File)**.

### Effects of plasma on virus infection

**Fig 4:**
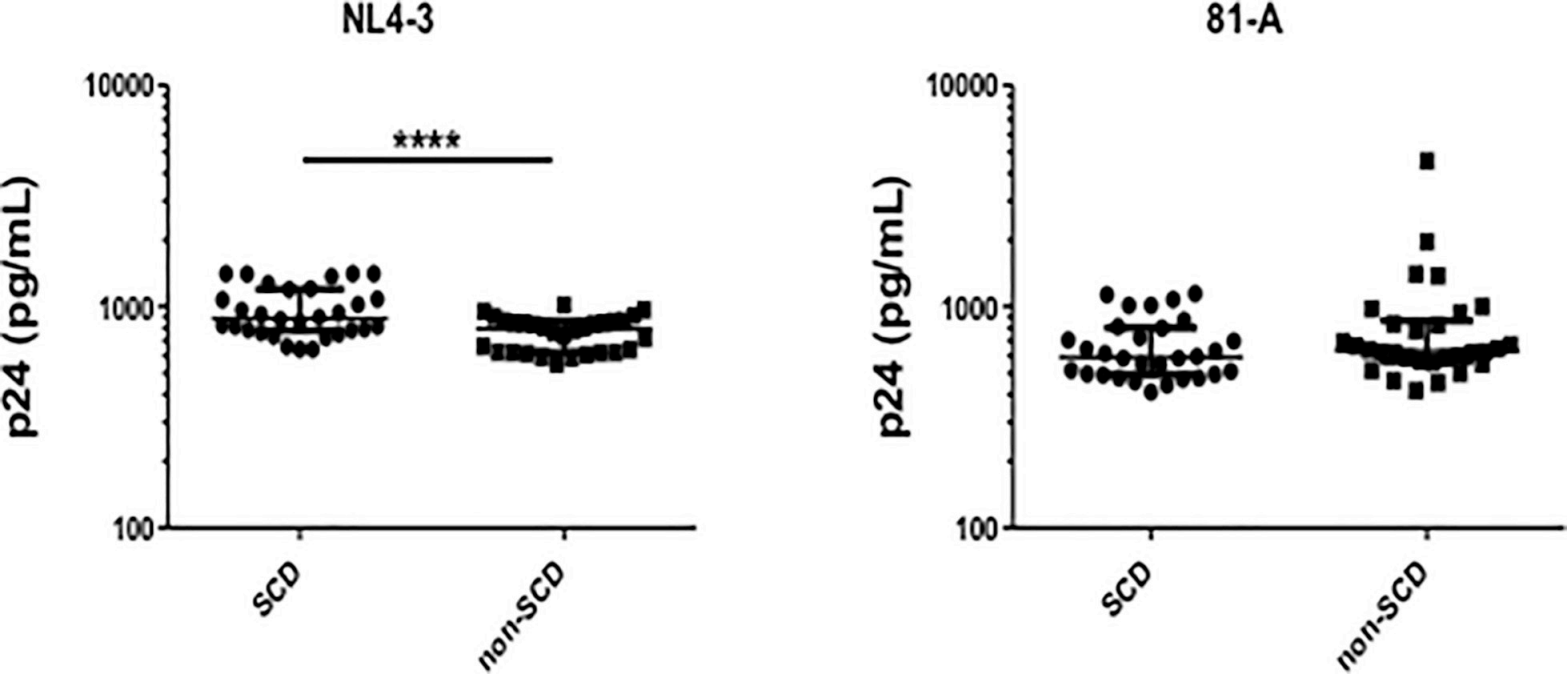
HIV Infectivity of Target MT-2 cells in the Presence of Plasma from SCD Patients or Plasma from Non-SCD Controls. MT-2 cells maintained at log phase growth were infected with either HIV NL4-3 (**Panel A**) or 81-A virus (**Panel B**) at MOI of 10^−2^ in cultures spiked with 20% plasma from SCD patients or non-SCD controls. Following 7-day infections supernatants were measured for p24 by ELISA. Mean and SEM are shown. * p<0.05.

Considering the differences in soluble factors between SCD and non-SCD controls, we hypothesized that some factors might be responsible for resistance to HIV. To determine the effects of plasma on infection, MT-2 cells were infected with either HIV NL4-3 or 81-A virus in the presence or absence of plasma from SCD patients or non-SCD controls. After X4 virus infection there was a significant increase in p24 production in the presence of SCD patient plasma compared to control plasma (**Fig 4A**). There was no significant difference in susceptibility of MT-2 cells to infection with R5 virus when infections were performed in the presence of SCD patient or control plasma (**Fig 4B**). These data do not show a protective effect of SCD patient plasma with infection of X4 or R5 virus.

## Discussion

In this study we retrospectively reviewed TSS data to determine if SCD patients were more or less likely than other transfused congenital anemia patients to become HIV seropositive during a high-risk period before the blood supply was consistently screened for HIV, and we performed *in vitro* experiments to investigate multiple potential mechanisms of HIV infection resistance in SCD. Although the TSS data demonstrated lower risk of HIV acquisition in transfused patients with SCD, the *in vitro* experiments did not reveal a mechanism of resistance to HIV infection.

The few reported studies investigating HIV in SCD have suggested a lower prevalence of HIV than in comparison populations[9, 17–19]. The risk of HIV acquisition in SCD may be modulated by delayed sexual maturation as older age of sexual debut has been reported in SCD [20]. However, other reports suggest improved HIV outcomes in SCD, indicating SCD pathophysiology may have the ability to ameliorate HIV infection. Bagasra et al. compared 18 HIV+ SCD patients (all HIV+ patients identified at 5 US SCD Centers) to 36 HIV+ non-SCD controls matched for age, race and gender. They reported 8 of 18 (44%) SCD cases were long-term non-progressors (LTNP, asymptomatic with low viral loads and CD4>500/mm without ART for at least 10 years) compared to 5 of 36 (13.9%) LTNP in controls, with an average follow up of 10 years (p = 0.0193). Death due to AIDS occurred in 5 of 18 (23%) HIV+ SCD patients vs. 22 of 36 (61%) of HIV+ controls [6]. The TSS presented a unique opportunity to investigate the susceptibility to transfusion transmitted HIV following a high-risk period when approximately 1% of blood units were HIV+ in some regions of the US[21]. Consistent with other epidemiological data, we found SCD was associated with lower odds of HIV acquisition compared to other highly transfused patients during this time. While we did not have access to additional HIV risk factors to compare between SCD and other patients, number of units transfused was associated with HIV status and age was not, suggesting transfusion was likely the primary HIV risk factor in these patients. The SCD patients received fewer blood products than other patients. However, after controlling for number of units received, congenital anemias other than SCD were independently associated with much higher odds of HIV acquisition in the TSS.

Considering the lower HIV acquisition rates in SCD patients, we sought to determine if there was an immunological component of SCD that might contribute to this outcome. We first investigated if SCD patients differed in their expression of markers associated with HIV entry into CD4^+^ T cells (CCR5, CXCR4) or cellular markers of activation (CD38 and HLA-DR). We demonstrated significantly lower expression of CCR5 in SCD patients, which was interesting as CCR5-tropic strains of HIV are generally associated with HIV transmission [22]. Despite the fact that SCD patients showed higher levels of immune activation as a consequence of their disease [23], these data pointed to a potential for lower levels of infection due to the decreased expression of the critical HIV co-receptor. Even so, the *in vitro* experiments did not show decreased susceptibility in SCD CD4^+^ T cells compared to controls, so the *in vivo* significance of lower CCR5 expression on CD4^+^ T cells in SCD is unclear.

We also sought to determine whether soluble factors found in plasma of SCD might mediate reduced susceptibility to HIV infection. As expected, many inflammatory cytokines were found to be elevated in SCD. Some of these cytokines have been implicated in inflammation-related pathogenesis in HIV infection in addition to inducing an activated state in CD4+ T cells, increasing the likelihood of those cells becoming infected[24]. Despite this inflammation, increased infection rates are not seen in SCD. Other studies have found that cytokines expressed during exposure may play a role in a protective phenotype. For example, Biasin et al. demonstrated a set of pro-inflammatory cytokines, including some we found elevated in SCD (IL-6, IL-12, TNF-α), were elevated in PBMCs and cervical mucosa of women highly exposed to HIV who did not seroconvert [25]. In addition, MIP-1α and MIP-1β, which were significantly higher in SCD patients, can bind HIV co-receptors to inhibit HIV fusion and entry. Finally, there were differences in cell adhesion molecules which may affect infection and cellular trafficking required to disseminate infection[26]. Despite these findings, we did not demonstrate a decrease in HIV infectivity in the presence of SCD plasma compared to control plasma and therefore, our *in vitro* experiments show that plasma itself had no detectable role in HIV susceptibility in the study population.

A recent publication by Kumari et al. demonstrated that increased iron export by ferroportin may restrict HIV infection in SCD via up-regulation of SAMHD1 [27]. In contrast, our data did not demonstrate resistance of SCD CD4^+^ T cells to HIV infection. The assays used in the two studies differed; the Kumari study used a single-cycle infection assay of PHA and IL-2 activated PBMCs. In our study CD8^+^-depleted, resting CD4^+^ T cells were infected with lab-adapted virus isolates. Given the activation of the target cells in the Kumari et al. study, it is possible that HIV restriction factors played a role in restricting infection of CD4^+^ T cells from SCD patients, and our assay lacking CD8^+^ T cells and exogenous activation did not detect this activity. A limitation of the current study is that levels of HIV restriction factors were not measured in the resting target CD4^+^ T cells. It would be worthwhile to replicate both viral infection techniques in a single patient population to determine which factors are important in demonstrating relative resistance of SCD PBMC populations to HIV infection.

Efforts to search for mechanisms that explain the lower rate of HIV infection in the SCD population are ongoing. These mechanisms should be explored in future studies to understand if other pathways may contribute to HIV infection resistance in SCD beyond the recently reported inhibition of HIV-1 reverse transcription triggered by inhibition of downstream pathways from a reduction in intracellular iron in SCD [27]. Of note, our study focused on CD4+ T cells as the target of HIV infection, and it is possible that monocytes or other cells from SCD patients may exhibit differential susceptibility to HIV infection. Unlocking these mechanisms of HIV resistance is important not only for understanding the interplay of SCD pathophysiology with potential infections in persons with SCD, but also for identification of potential therapeutic targets for HIV in general.

## Supporting information

S1 File S1A Table and S1B Table.
(DOCX)

S2 File Minimal dataset. (XLSX)

S1 Fig Comparison of HIV Co-receptors, CCR7 and Activation Markers between SCD Patients Treated or Not with Hydroxyurea. Comparison of HIV co-receptors (CCR5, CXCR4), CCR7 and activation markers (CD38 and HLA-DR) between SCD patients treated or not with hydroxyurea (HU). Bars represent means +/- SEM. *p<0.05.
(TIFF)

S2 Fig HIV Infectivity of PBMCs from SCD Patients Treated or Not with Hydroxyurea. Comparison of p24 after infection of NL4-3 (panel A) and 81-A (panel B) in CD8-depleted PBMC from SCD patients and non-SCD controls at MOI of 10^−2^, 10^−3^ and 10^−4^.
(TIFF)
