# Supplementary material for: Influence of sickle cell disease on susceptibility to HIV infection
Source: PLoS One. 2020 Apr 8;15(4):e0218880. doi: 10.1371/journal.pone.0218880 (PMC7141606; doi:10.1371/journal.pone.0218880)
Supplement: S1 File — S1A Table and S1B Table. (DOCX) [file pone.0218880.s001.docx]

**S1 Table A: Literature Reporting HIV Prevalence in Sickle Cell Disease**

| **Reference** | **Year** | **HIV+ SCD** | **HIV+ Control Population** |
| --- | --- | --- | --- |
| Ouattara^1^ | 1988 | 15 of 67 (22.4%) transfused SCD children (Ivory Coast) | 2 of 320 (0.62%) NON-transfused non SCD children at same hospital/time period |
| Wawero^2^ | 1989 | 0 of 198 transfused SCD children (Kenya) | 3 of 54 (7.4%) transfused non SCD children at same hospital/time period |
| Castro^3^ | 1990 | 0 of 88 transfused SCD (US) | No true control population in study. Authors compared HIV in SCD to estimated 70-80% transfused hemophilia patients infected with HIV in same time period |
| Wawero^4^ | 1992 | 1 of 44 (2.3%) transfused SCD (Kenya) | 3 of 4 (75%) transfused non SCD at same hospital/time period |
| Diagne^5^ | 2000 | 0 of 155 screened SCD patients (Senegal) | None |
| Ocak^6^ | 2006 | 0 of 210 transfused SCD patients (Turkey) | None |
| Dokekias^7^ | 2009 | 0 of 112 screened transfused SCD patients (Congo-Brazzaville) | None |
| Batina Agasa ^8^ | 2010 | 1 of 140 (0.7%) transfused SCD (Democratic Republic of Congo) | 184 of 3390 (5.4%) blood donors in same geographical area (p=0.022) |
| Moura Neto^9^ | 2011 | 11 of 1415 adult SCD (0.8%) (Brazil) | No true control population, but similar HIV prevalence as HIV reported in general population of same geographical area |
| Nouraie ^10^ | 2012 | 94 HIV+ diagnosis with SCD diagnosis of 6517 (1.4%) discharges in US national hospital discharge data of African Americans >20 from 1997-2009 (US) | 13,256 HIV+ diagnosis without SCD diagnosis of 416,914 (3.2%) discharges in same database (p>0.0001) |
| Diarra^11^ | 2013 | 2 of 133 (1.5%) SCD prospectively screened for HIV (Mali) | None |

**S1 Table B: Cytokines and Chemokines Tested in SCD and non-SCD Controls**

| Cytokine | Median (Range) Value SCD | Median (Range) Value non-SCD | FDR Corrected p value |
| --- | --- | --- | --- |
| IL-10 | 9.3 (3.6-24.1) | 8.0 (3.4-21.9) | 0.40 |
| IL-13 | 2.4 (1.0-15.3) | 2.9 (0.9-13.2) | 0.57 |
| IL-4 | 14.7 (4.0-52.8) | 16.9 (6.2-44.5) | 0.79 |
| IL-5 | 2.0 (0.7-5.2) | 1.7 (0.6-15.8) | 0.19 |
| MMP-9 | 109,000 (17000-378000) | 56,400 (30800-245000) | 0.08 |
| TRAIL | 23.8 (1.3-71.7) | 23.9 (1.3-76.6) | 0.91 |
| Fractalkine* | 115 (18.1-215) | 82.5 (38.6-178) | 0.02 |
| MIP-1α* | 10.2 (5.3-259) | 7.8 (4.0-64.6) | 0.02 |
| MIP-3α* | 16.9 (10.7-47.6) | 14.8 (8.3-33.1) | 0.04 |
| IP-10 | 292 (103-1330) | 241 (113-494) | 0.16 |
| MCP-1 | 172 (19.6-364) | 164(60.7-521) | 0.37 |
| MDC | 929(596-2920) | 884 (436-2920) | 0.56 |
| MIP-1β* | 18.7 (25.5-826) | 12.3 (19.5-497) | 0.01 |
| HCC-1 | 1970 (1250-4080) | 1760 (1140-2980) | 0.24 |
| MIP-3β* | 114 (56.3-311) | 77.1 (44.4-744) | 0.003 |
| MIG* | 952 (343-2780) | 539 (176-2210) | 0.002 |
| Lymphotactin | 97.2 (67.3-1380) | 106 (41.3-308) | 0.41 |
| 6CKINE | 534 (123-1330) | 398(175-996) | 0.08 |
| BCA-1* | 15.4 (5.1-36.4) | 20.2 (7.7-74.9) | 0.008 |
| CTACK | 777 (366-1450) | 781 (320-1350) | 0.59 |
| Eotaxin-2 | 306 (43.3-697) | 247 (24.9-1100) | 0.89 |
| SDF-1α+β* | 244 (243-4410) | 3910 (244-8590) | 0.001 |
| Fibrinogen* | 1,560,000(566,000-3,900,000) | 1,270,000 (913,000-2,580,000) | 0.03 |
| vWF* | 14,700 (3,770-55,100) | 6,140 (2620-31,200) | 0.00001 |
| GM-CSF | 19.1 (3.3-236) | 22 (2.9-1240) | 0.99 |
| FGF-2 | 156 (55.9-823) | 116 (56.4-924) | 0.12 |
| TGF-α* | 8.9 (2.2-47.7) | 4.5 (1.2-263) | 0.001 |
| VEGF | 630 (266-5400) | 521 (234-5320) | 0.45 |
| IFN-γ | 9.9 (1.6-23.1) | 10.2 (1.8-19.9) | 0.66 |
| IL-12p70* | 2.6 (0.9-5.0) | 1.9 (0.9-4.4) | 0.04 |
| IL-1β* | 3.9 (0.7-25.5) | 2.3 (0.9-5.9) | 0.002 |
| IL-2 | 2.8 (0.6-5.4) | 2.5 (0.6-15.8) | 0.88 |
| IL-21* | 2.6 (1-6.5) | 1.5 (0.5-3.5) | 0.003 |
| IL-23 | 103 (26.9-289) | 69.1 (24.2-319) | 0.07 |
| IL-6* | 2.1 (0.6-4.4) | 1.1 (0.6-3.9) | 0.004 |
| IL-7 | 5.7 (2.5-11) | 5.0 (2.9-11.9) | 0.21 |
| IL-8* | 3.8 (1.7-90.9) | 2.4 (0.9-7.8) | 0.001 |
| TNF-α* | 6.1 (4.1-12) | 3.9 (2.1-5.8) | <0.0005 |
| IFN-α2 | 89.1 (10.1-2460) | 64.5 (25.9-214) | 0.16 |
| IL-15 | 5.5 (0.7-38.9) | 3.0 (0.7-49.5) | 0.41 |
| IL-17A | 7.8 (4.8-612.1) | 6.0 (4.2-546.3) | 0.25 |
| ITAC* | 309(26.1-1130) | 53.0 (19.2-278) | 0.00001 |
| MIP-1δ | 1800 (1080-6140) | 1680 (296-4140) | 0.89 |
| MPO* | 132 (19.7-393) | 38.4 (16.2-101) | <0.0005 |
| sICAM* | 588 (56.4-1390) | 168 (71-979) | 0.0001 |
| sVCAM* | 572 (323-1210) | 499 (331-794) | 0.01 |
| Adipsin | 4190 (850-12300) | 4190 (850-9810) | 0.81 |
| AGP* | 1,730,000 (929000-2360000) | 1,230,000 (825000-1940000) | 0.001 |
| CRP* | 124000 (1630-98200) | 2,270 (353-29100) | 0.00001 |
| Fetuin | 133000 (73000-188000) | 123000 (90000-192000) | 0.70 |
| L-Selectin* | 621 (322-988) | 723 (465-1040) | 0.01 |
| PF4/CXCL4 | 5510 (1080-12200) | 4520 (1450-14200) | 0.18 |
| SAP* | 5250 (2380-9120) | 6520 (3190-8420) | 0.036 |

1. Ouattara SA, Gody M, Rioche M, et al. Blood transfusions and HIV infections (HIV1, HIV2/LAV2) in Ivory Coast. *J Trop Med Hyg* 1988; **91**(4): 212-5.

2. Waweru SEN MJ, Kinuthia DM, Kitoyi GW. Absence of HIV seropositivity in children with sickle cell anemia at Kenyatta Nationao Hospital. *International Conference AIDS* 1989; (5): 248.

3. Castro O, Saxinger C, Barnes S, Alexander S, Flagg R, Frederick W. Prevalence of antibodies to human immunodeficiency virus and to human T cell leukemia virus type I in transfused sickle cell disease patients. *J Infect Dis* 1990; **162**(3): 743-5.

4. Waweru SEN OS, Muniu E, Libondo J. Persistent low HIV infection in transfused sicklers as compared to high HIV infection in children presenting with other types of anaemia requiring blood transfusion. *Nairobi J Med* 1992; **8**: 26.

5. Diagne I, Soares GM, Gueye A, et al. [Infections in Senegalese children and adolescents with sickle cell anemia: epidemiological aspects]. *Dakar Med* 2000; **45**(1): 55-8.

6. Ocak S, Kaya H, Cetin M, Gali E, Ozturk M. Seroprevalence of hepatitis B and hepatitis C in patients with thalassemia and sickle cell anemia in a long-term follow-up. *Arch Med Res* 2006; **37**(7): 895-8.

7. Dokekias AE, Ossini LN, Tsiba FO, Malanda F, Koko I, De Montalembert M. [Blood transfusion assessment to 112 homozygous sickle-cell disease patients in university hospital of Brazzaville]. *Transfus Clin Biol* 2009; **16**(5-6): 464-70.

8. Batina Agasa S, Dupont E, Kayembe T, et al. Multiple transfusions for sickle cell disease in the Democratic Republic of Congo: the importance of the hepatitis C virus. *Transfus Clin Biol* 2010; **17**(4): 254-9.

9. Neto JP, Lyra IM, Reis MG, Goncalves MS. The association of infection and clinical severity in sickle cell anaemia patients. *Trans R Soc Trop Med Hyg* 2011; **105**(3): 121-6.

10. Nouraie M, Nekhai S, Gordeuk VR. Sickle cell disease is associated with decreased HIV but higher HBV and HCV comorbidities in US hospital discharge records: a cross-sectional study. *Sex Transm Infect* 2012.

11. Diarra AB, Guindo A, Kouriba B, et al. [Sickle cell anemia and transfusion safety in Bamako, Mali. Seroprevalence of HIV, HBV and HCV infections and alloimmunization belonged to Rh and Kell systems in sickle cell anemia patients]. *Transfus Clin Biol* 2013; **20**(5-6): 476-81.
