# Supplementary figures and images for: Influence of sickle cell disease on susceptibility to HIV infection
Source: PLoS One. 2020 Apr 8;15(4):e0218880. doi: 10.1371/journal.pone.0218880 (PMC7141606; doi:10.1371/journal.pone.0218880)

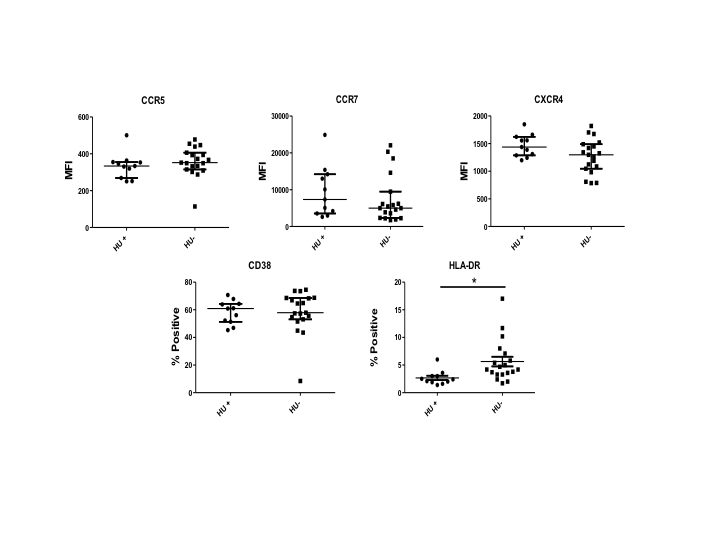

Supplement: S1 Fig — Comparison of HIV co-receptors (CCR5, CXCR4), CCR7 and activation markers (CD38 and HLA-DR) between SCD patients treated or not with hydroxyurea (HU). Bars represent means +/- SEM. *p<0.05. (TIFF) [file pone.0218880.s003.tiff]

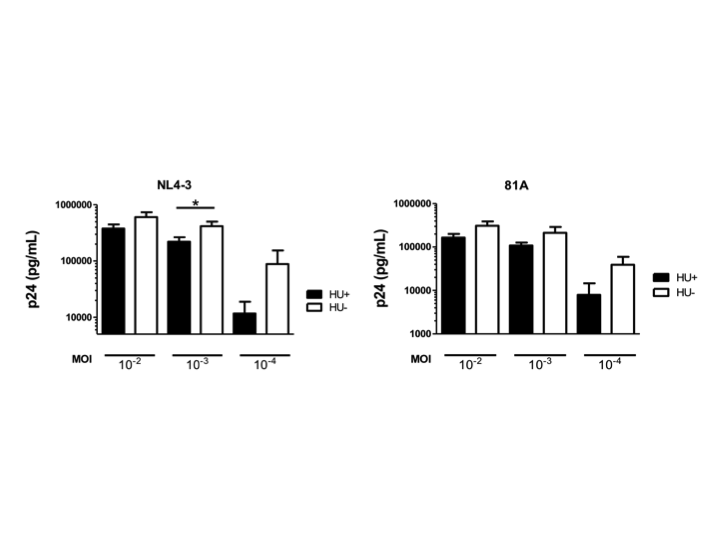

Supplement: S2 Fig — Comparison of p24 after infection of NL4-3 (panel A) and 81-A (panel B) in CD8-depleted PBMC from SCD patients and non-SCD controls at MOI of 10−2, 10−3 and 10−4. (TIFF) [file pone.0218880.s004.tiff]
